# Grief is a family affair: examining longitudinal associations between prolonged grief in parents and their adult children using four-wave cross-lagged panel models

**DOI:** 10.1017/S0033291723001101

**Published:** 2023-11

**Authors:** L. I. M. Lenferink, M. O'Connor

**Affiliations:** 1 Department of Psychology, Health, & Technology, Faculty of Behavioural, Management, and Social Sciences, University of Twente, Drienerlolaan 5, 7522 NB Enschede, the Netherlands; 2 Department of Clinical Psychology and Experimental Psychopathology, Faculty of Behavioral and Social Sciences, University of Groningen, Groningen, the Netherlands; 3 Department of Clinical Psychology, Faculty of Social Sciences, Utrecht University, Heidelberglaan 1, 3584 CS Utrecht, the Netherlands; 4 Department of Psychology, Unit for Bereavement Research, Aarhus University, Aarhus, Denmark; 5 The Danish National Center for Grief, Copenhagen, Denmark

**Keywords:** Bereavement, child, dyads, families, loss, parent, prolonged grief

## Abstract

**Background:**

Losing a parent or spouse in adulthood may result in prolonged grief disorder (PGD) symptoms. PGD levels in parents may affect PGD levels in their adult offspring and the other way around. However, research on transmission of PGD in parent–child dyads is lacking. Consequently, we aimed to examine temporal associations between PGD levels in parent and adult children.

**Methods:**

In doing so, we analyzed longitudinal self-report data on PGD levels (using the PG-13) assessed at 2, 11, 18, and 26 months after loss in 257 adult parent–child dyads from Denmark. Cross-lagged panel modeling was used for data-analyses.

**Results:**

Changes in PGD levels in parents significantly predicted PGD levels in adult children, but not vice versa. Small through moderate cross-lagged effects (*β* = 0.05 through 0.07) were found for PGD levels in parents predicting PGD levels in adult children at a subsequent time-point. These cross-lagged effects were found while taking into account the association between PGD levels in parents and adult children at the same time-point as well as the associations between the same construct over time and relevant covariates.

**Conclusions:**

Pending replication of these findings in clinical samples and younger families, our findings offer tentative support for expanding our focus in research and treatment of PGD from the individual to the family level.

A prolonged grief disorder (PGD) is characterized by intense and long-lasting grief reactions, such as separation distress, that interfere with daily life of people who lost a significant other at least 6 months earlier (World Health Organization, 2022). A pooled prevalence rate of 9.8% for probable PGD has been found in adults who experienced a non-violent loss (Lundorff, Holmgren, Zachariae, Farver-Vestergaard, & O'Connor, 2017). Higher PGD severity has been found in women, people who are lower educated, more recently bereaved people, and people whose loved one died suddenly and/or violently (Heeke, Kampisiou, Niemeyer, & Knaevelsrud, 2019; Lobb et al., 2010). There is also a body of research indicating that losing a member of the nuclear family, such as a spouse or parent, is associated with higher PGD severity than losing a more distantly related family member or friend (Heeke et al., 2019; Lobb et al., 2010).

It has been repeatedly shown that children whose mothers or fathers are depressed are likely to experience elevated psychopathological problems (Brennan, Hammen, Katz, & Le Brocque, 2002; Goodman et al., 2011). Similar findings have been found for the transmission of posttraumatic stress symptomatology in parent–child dyads after trauma exposure (Dyb, Jensen, & Nygaard, 2011; Lambert, Holzer, & Hasbun, 2014; Schwerdtfeger & Goff, 2007). After a death of a parent, children often do not grieve alone. Based on findings in the field of depression and posttraumatic stress, it is likely that PGD levels in parentally bereaved people are influenced by the PGD levels in their remaining parent.

Theoretical and empirical work offers several hypotheses on how PGD levels may rise in children of people with elevated PGD symptoms. Firstly, transmission of psychopathology in parent–child dyads may occur because of the shared genetics (Rutter, 2007). To illustrate this, heritability of susceptibility for developing posttraumatic stress symptoms, and even exposure to traumatic events, have been found in prior research among trauma-exposed samples (Kremen, Koenen, Afari, & Lyons, 2012); this may also apply to PGD transmission within biological parent–child dyads. From a neurobiological perspective, PGD has been conceptualized as a disorder of reward (e.g. craving for the deceased while simultaneously avoiding emotional pain related to the loss), which shows some similarities with other disorders of reward, such as addiction (Kakarala et al., 2020; O'Connor et al., 2008). Dysregulation in brain reward systems seems to some extent heritable (Dichter, Damiano, & Allen, 2012), which may partly explain why PGD is transmissible from parent to child.

Secondly, environmental factors are likely to impact the interactions between PGD levels in parents and children. Drawn from theories about social learning (Bandura, 1977) and interpersonal theories of depression and posttraumatic stress (Coyne, 1976; Maercker & Horn, 2013), being exposed to negative cognitions and behaviors from a significant other may elucidate negative cognitions and behaviors through modeling behavior. When applying this to PGD in parent–child dyads, witnessing intense grief reactions in your child after losing your spouse, or witnessing this in your remaining parent after losing one of your parents, may contaminate your own grief reactions. Other environmental factors that may relate to severity of grief reactions in parent–child dyads are family functioning (Gelcer, 1983) and family dynamics (Bowen, 1978). Family functioning includes among others the level of adaptability to change, level of conflict, and quality of communication within families (Alderfer et al., 2008). The death of a nuclear family member likely brings about dynamic changes within the family, which may revive former dysfunctional patterns of family functioning, such as insecure attachment styles (Krause & Haverkamp, 1996). In a similar vein, stemming from Bowen's family systems theory (Bowen, 1978; Ceja & Gasbarrini, 2018), parents may consciously as well as unconsciously project their own emotions, such as intense sadness after loss (e.g. originating from anxious attachment), onto their children. Those belonging to families with healthy family functionality and dynamics may cope more adaptively with stressful life changes (such as a death), conflicts, and negative emotions than families with low family functionality (Ceja & Gasbarrini, 2018; Çankaya & Alan Dikmen, 2022; Kissane et al., 1996; Thomas, Liu, & Umberson, 2017).

While prior work suggests that depression and posttraumatic stress are partly transmissible between parents and children, empirical evidence for intergenerational grief within parent–child dyads after loss is still limited, with one notable exception. A study among bereaved families with a refugee background indicated that elevated PGD levels in parents were positively associated with children's (aged 11–17) behavioral, emotional, and social problems (Bryant et al., 2020). This was, however, a cross-sectional study, whereby PGD levels were assessed in parents, but not in their children. This study therefore precludes to draw conclusions about to what extent grief reactions in parents precede grief reactions in children or vice versa.

Examining the interplay between grief reactions within families is urgently needed to inform theorizing about intergenerational effects of bereavements. Furthermore, these findings can ultimately improve treatment effects for PGD, for example, such that when PGD interacts between parents and their offspring, using a more systematic approach by targeting PGD in the family context in treatment is of utmost important for alleviating PGD. Accordingly, our main aim of the current study was to provide first insights into reciprocal associations between PGD levels in dyads of spousal bereaved people and adult children. Based on prior research on temporal associations between posttraumatic stress reactions in parent–child dyads (Egberts, van de Schoot, Geenen, & Van Loey, 2018; Honda et al., 2019; Silverstein et al., 2022), we expected that changes in PGD levels in parents preceded changes in PGD levels in adult children (and not vice versa).

## Methods

### Participants and procedures

Data for the present study were drawn from The Aarhus Bereavement study (TABstudy) (Harris, Brookman, & O'Connor, 2021; Lundorff, Bonanno, Johannsen, & O'Connor, 2020; Maccallum, Lundorff, Johannsen, Farver-Vestergaard, & O'Connor, 2021). This on-going study aims to collect data for at least a decade to examine the severity, etiology, course, and treatment of reactions to loss by using self-report survey data on physical and mental health. Participants were identified through the Danish Civil Registration System (DCRS) which provided information on bereaved individuals who lost their spouses. All people who lived in the metropolitan area of Aarhus in Denmark and lost a spouse between January 2017 and March 2018 were sent a condolence letter approximately one month post loss. In this letter, they were informed that they would be invited by telephone for participation in this study 2 months post-loss. Participants were also invited to share information about the research with their adult children and to offer contact information of their children to the researchers, if the children were interested in being a part of the study. Participation consisted of completing surveys online or with paper-and-pencil.

In total, 2006 spousal bereaved people were sent a condolence letter, of which 542 (27%) were undetectable by phone for the following reasons: (1) phone number was unavailable (*n* = 253), (2) no contact after multiple phone calls (*n* = 190), and (3) declined participation after receiving the condolence letter (*n* = 99). Of the 1464 contacted spousal bereaved people, 986 (67%) were willing to participate [for more information see Harris et al. (2021); Maccallum et al. (2021)]. Adult children of these bereaved spouses were also invited to participate. In total, 257 families, including a spousal bereaved person and at least one adult child, participated. In some cases, multiple children from one parent participated (18% of cases in this sample). We only analyzed data from parent–child dyads in this study. Following prior research (Silverstein et al., 2022), we randomly selected which child data we included in our analyses in case multiple adult children participated from one spousal bereaved person.

The first assessment [referred to as wave 1 (W1)] took place 2 months post-loss (data were collected between March 2017 and May 2018). Wave 2 (W2) was conducted 11 months post-loss (data were collected between December 2017 and February 2019). Wave 3 (W3) was completed 18 months post-loss (data were collected between July 2018 and September 2019). Wave 4 (W3) was completed 26 months post-loss (data were collected between March 2019 and May 2020). While in between W1 and W2 another assessment toke place 6 months post-loss, we excluded this wave in this study, in order to make sure that the time-interval between each wave was equal [which is crucial for accuracy in estimating parameters in time-dependent models (Baribeau et al., 2022)]. The overall time-interval between all waves was 8 months.

### Measures

PGD levels in parents and adult children at all waves were measured using the Prolonged Grief Disorder-13 (PG-13) (Prigerson et al., 2009). This13-item scale includes two dichotomized items regarding the duration and impairment of grief reactions. The remaining 11 items assesses cognitive, behavioral, and emotional symptoms of precursor criteria for PGD^†^^1^. An example item is ‘In the past month, how often have you tried to avoid reminders that the person you lost is gone?’ People answered these items on five-point scales ranging from 1 (not at all) to 5 (several times a day or overwhelmingly). The 11 items were summed to represent PGD severity, with higher scores reflecting more pervasive PGD severity. A preliminary cut-off score of ⩾35 has been recommended for probable PGD caseness (Pohlkamp, Kreicbergs, Prigerson, & Sveen, 2018). The PG-13 have shown to yield sound psychometric properties in prior research (Pohlkamp et al., 2018; Prigerson et al., 2009). Cronbach's *α* levels were 0.90 for W1, 0.92 for W2, 0.91 for W3, and 0.89 for W4.

### Statistical analysis

Cross-lagged panel analyses were performed using Mplus Version 8.4 (Muthén & Muthén, 1998), to examine to what extent PGD levels in parents predict PGD levels in children over time (or vice versa) across bereaved families. In doing so, we relied on dyad data on PGD levels collected across four waves. The cross-lagged panel model included cross-lagged paths, in which we regressed the PGD levels in parents assessed at a certain wave on the PGD levels in adult children assessed at a preceding wave, and the other way around; so PGD levels in adult children assessed at a certain wave were regressed on PGD levels in parent assessed at a preceding wave. Autoregressive paths were included, which takes into account the correlation between a construct over time. In our case, this was included for the associations between PGD levels in adult children at W1, W2, W3, and W4 and the associations between PGD levels assessed in parents across these four waves. Lastly, we also took the associations between adult child and parent PGD levels assessed at the same wave into account (e.g. adult child PGD at W1 with parent PGD at W1).

In subsequent steps of the analyses, we constrained parameters, as described below, to be equal in order to select the most parsimonious model (Little, 2013). In doing so, we compared the fit of the unconstrained model (model 1), with the fit of a model in which we constrained the autoregressive paths to be equal for child as well as parent PGD levels (model 2). In the next step, we further constrained the model by assuming the cross lagged effects to be equal across the waves (model 3). Lastly, we also included constraints on the associations between the constructs at the same wave by assuming these to be equal over time (model 4).

Selection of most parsimonious model was based on the following fit statistics and guidelines: comparative fit index (CFI) and Tucker–Lewis index (TLI) values >0.90 are indicative of acceptable fit and >0.95 excellent fit, root mean square error of approximation (RMSEA) values <0.10 reflect acceptable fit and <0.06 excellent fit, standardize root mean square residual (SRMR) values <0.08 reflect acceptable fit and <0.06 excellent fit, and for nested models smaller values for Akaike information criterion (AIC) and sample size-adjusted Bayes information criterion (SA-BIC) are preferred (Baribeau et al., 2022). After selecting the most parsimonious model, we also included the following known risk factors for PGD as covariates in our model by regressing PGD at W1 on these covariates: gender of parent, child, and deceased (0 = man, 1 = woman) and educational level of parent and child (0 = other than college/university, 1 = college/university). Lastly, we also tried to include random intercepts into the cross-lagged models (cf. Keijsers, 2016), but none of the models converged. Our limited sample size likely resulted in convergence issues; we therefore estimated cross-lagged models without random intercepts. Full information maximum likelihood estimation was used to handle missing data on PGD outcomes. Two-tailed tests were run with *α* = 0.05.

## Results

### Characteristics of the participants

Table 1 shows the characteristics of the sample. Three out of five adult children and three-quarter of the parents identified themselves as woman. Mean age was 44 in adult children and 71 in parents. About half of the adult children completed college or university and one-third of the parents did so. In three out of five cases, the death was caused by cancer. Regarding probable PGD caseness, 11% of adult children scored above cut-off for PGD at W1, 7% at W2, 5% at W3, and 1% at W4. For parents these rates were 19, 11, 8, and 3%, respectively.

**Table 1.** Characteristics of the participants

	Adult children (*N* = 257)	Parents (*N* = 257)	Total (*N* = 514)
Sex of participant, *N* (%)	159 (62)	186 (72)	345 (67)
Sex of deceased, *N* (%)	–	–	186 (72)
Age, M (s.d.)	44.16 (9.91)	70.76 (8.71)	57.51 (16.25)
Years of cohabiting for parents, M (s.d.)	n/a	44.16 (9.91)	n/a
College/university, *N* (%)	124 (49)	65 (27)	189 (38)
Income, *N* (%)			
Salary	215 (84)	39 (16)	254 (50)
Old age pension	8 (3)	197 (79)	205 (41)
Other	32 (13)	14 (5)	46 (9)
Cause of death, *N* (%)	–	–	
Cancer			148 (60)
Cardio vascular diseases			26 (11)
Old age			10 (4)
Dementia			5 (2)
Accident			3 (1)
Suicide			1 (0)
Diabetes			2 (1)
Other			53 (21)

*Note*. - = The responses were equal for parents and children; n/a, not applicable. The following responses were missing: for education level 12 responses were missing in parents and five in adult children; for income seven were missing in parents and two in adult children; for cause of death nine responses were missing.

### Associations between PGD levels in parents and adult children

Table 2 shows means, standard deviations, and score ranges of, and correlations among, PGD levels in adult children and parents at all waves. PGD levels in adult children were significantly, positively, and strongly associated across each wave. Similar associations were found for PGD levels in parents across the waves. Associations between PGD levels in adult children and parents over time were significant, positive, and small through moderate.

**Table 2.** Means, standard deviations, and bivariate associations between PGD in adult children and PGD in parents across the four waves (*N* = 514)

	Descriptive statistics				PGD in adult children				PGD in parents			
Construct	*N*	M	s.d.	Observed range	W1	W2	W3	W4	W1	W2	W3	W4
PGD in adult children												
W1	252	22.10	8.88	11–52		0.846	0.749	0.735	0.354	0.306	0.285	0.279
W2	216	19.00	7.70	11–44			0.886	0.846	0.297	0.275	0.259	0.269
W3	186	17.84	6.72	11–42				0.876	0.352	0.323	0.314	0.296
W4	138	16.78	5.83	11–43					0.336	0.389	0.306	0.352
PGD in parents												
W1	248	26.68	8.14	11–50						0.768	0.726	0.703
W2	233	23.44	8.37	11–48							0.781	0.780
W3	204	21.50	7.46	11–45								0.887
W4	186	20.24	6.96	11–46								

M, mean; PGD, prolonged grief disorder; s.d., standard deviation; W, wave.

*Note.* All correlations are significant at *p* < 0.001.

**Figure. 1.**
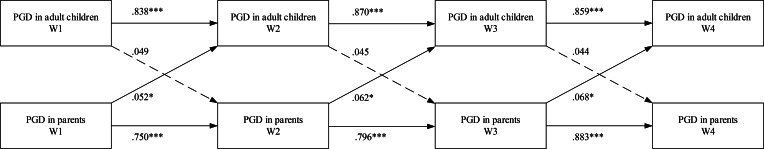
Standardized auto-regression and cross-lagged paths between PGD levels over time in parent–child dyads (*N* = 257). *Note*. Associations between PGD in adult children and parents at the same wave are not shown. Dashed lines present non-significant paths. PGD, prolonged grief disorder; W, wave. ***p < 0.001, *p < 0.05.

Online Supplementary Table S1 shows the fit indices of the cross-lagged models. The model including constrained autoregressive and cross-lagged paths showed the best fit as evidenced by an excellent CFI and acceptable TLI value, the lowest RMSEA value and acceptable SRMR value, and the smallest SA-BIC and AIC values. Standardized estimates of the most optimal model are shown in Fig. 1 (for unstandardized estimates see online Supplementary Table S2). PGD levels in parents significantly predicted PGD levels in adult children at a subsequent wave. However, PGD levels in adult children did not predict PGD levels in parents at a subsequent wave. When adding covariates to this model results did not change meaningfully (i.e. same paths were significant^2^).

## Discussion

This is the first study examining reciprocal associations between PGD levels in parent–child dyads. In doing so, we analyzed PGD data from 257 adult parent–child dyads who lost their spouse or parent due to (in vast majority of the cases) natural causes (e.g. illness). Parents and their adult children completed PGD measures 2, 11, 18, and 26 months post-loss. The time-intervals cover acute PGD levels (i.e. people bereaved less than 6 months earlier) and long-term PGD severity, which enabled us to examine reciprocal associations between PGD levels in parents and children over the first 2 years of bereavement.

Our main finding was that PGD levels in parents predicted PGD levels in adult children, while taking into account the association between PGD levels in parents and adult children at the same time-point as well as the associations between the same construct over time and relevant covariates. PGD levels in adult children did not predict PGD levels in parents. These findings accord with research in non-bereaved samples indicating that parental depression or PTSD are related to increased psychopathology levels in children (Egberts et al., 2018; Lieb, Isensee, Höfler, Pfister, & Wittchen, 2002; Silverstein et al., 2022). These prior dyad studies predominantly included minor children. While our sample included adults only, we were still able to detect small through moderate cross-lagged effects (Orth et al., 2022). Assuming that these intergenerational effects are partly explained by environmental factors (Bandura, 1977; Bowen, 1978; Coyne, 1976; Maercker & Horn, 2013), it is plausible that these effects are even more profound in families with minor children [who share (greater parts of) their household with their parents] than in families with adult children.

That we were still able to detect that parental PGD is predictive of PGD levels in adult children might be (partly) explained by the composition of this sample. The majority of this sample were bereaved by illness. Most parents and adult children provided care to the deceased prior to death and, thus, likely faced pre-existing family patterns that were already formed when they were a young family. It is common that more time is spent with family members in the palliative phase of a nuclear family member as well as in the acute grief phase, which may partly explain why in adulthood it is possible to find effects for children mirroring parental grief.

If similar or even greater cross-lagged effects are found in future studies including bereaved parents and minor children who are distressed post-loss, this may suggest that targeting PGD in parents is a valuable addition to PGD treatment for bereaved children. Indeed, treatment for childhood PGD that includes parental involvement has shown to be effective (Boelen, Lenferink, & Spuij, 2021; Sandler et al., 2010). More specifically, Sandler et al. (2010) showed that the group-based Family Bereavement Program, targeted at enhancing effective parenting skills (e.g. anger management techniques and listening skills) and improving coping skills in children (e.g. good communication and problem solving skills) (Ayers et al., 2013), alleviated grief in parentally bereaved youth in the short and long run. More recently, individual grief-specific cognitive-behavioral therapy in bereaved minors with PGD, including paralleled counseling sessions for parents/caretakers focused on supporting their child and strengthen the parent–child relationship, yielded large within-group effects. Small to medium between-group effects for PGD were found compared to supportive counselling immediately after treatment and at 3, 6, and 12 months follow-up (Boelen et al., 2021).

This seminal work on treatment for childhood PGD with parental involvement suggests that parental involvement seems effective, however, research comparing effects of childhood PGD treatment with and without parental involvement is, to the best of our knowledge, lacking. Furthermore, the potential impact of the extent and type of parental involvement on treatment outcomes for PGD in children still remains to be studied. Future research may also benefit from exploring the efficacy of other family treatment approaches, such as Bowen's family systems approach or a conceptual framework of attachment to explain parent–child interactions (Bowen, 1978; Ceja & Gasbarrini, 2018; Krause & Haverkamp, 1996). For the latter, it should be noted that empirical evidence supporting its effectiveness and working mechanisms is still very much needed (Calatrava, Martins, Schweer-Collins, Duch-Ceballos, & Rodríguez-González, 2022; Suomi, Evans, Rodgers, Taplin, & Cowlishaw, 2019).

While our findings offer first tentative support that PGD is transmissible from parent through adult child, we were not able to examine why this might be the case. Insights in malleable factors [such as problematic parent–child communication (Zhen, Yao, & Zhou, 2022), poor conflict management styles (Chiariello & Orvaschel, 1995), deficient emotion-regulation strategies (Lougheed, Brinberg, Ram, & Hollenstein, 2020), or maladaptive coping styles (Krause & Haverkamp, 1996)] potentially mediating associations between parent–child transmission of PGD may provide input for further refinement of PGD treatment.

Several study limitations should be noted. First, the majority of our sample did not meet criteria for probable PGD. About one out of 10 adult children and one out of five parents reported elevated PGD levels at W1 and these PGD rates seem to decrease over time. Caution is warranted when generalizing our findings to clinical samples. Second, data collection for this study started before PGD criteria entered the DSM-5-TR and ICD-11 (American Psychiatric Association, 2022; World Health Organization, 2022). The PGD instrument that we used does not capture all of these most recent PGD criteria. Our results therefore may not necessarily generalize to studies using these updated criteria (Lenferink, Boelen, Smid, & Paap, 2019). Third, it was not possible to include random intercepts in our statistical models, likely due to our limited sample size. Consequently, we were unable to make a distinction between within-dyad and between-dyad differences (Orth, Clark, Donnellan, & Robins, 2021). Including random intercepts in future studies likely provide stronger evidence for potential causal linkage between PGD in parent and their offspring (Keijsers, 2016).

Taken together, this is the first study examining reciprocal associations between PGD levels in adult–child dyads after loss. Within the first 2 years after loss, we found that changes in PGD levels in parents seem to proceed changes in PGD levels in adult children. When replicating our findings in clinical samples as well as younger families, this offers preliminary support for transmission of PGD from parents to children, which points to the need of expanding the focus of PGD research and treatment from the individual level to family level.

## Supporting information

Lenferink and O'Connor supplementary material Lenferink and O'Connor supplementary material

## Footnotes

† The notes appear after the main text.

## Notes

1 Data collection for this study started before the introduction of PGD criteria in ICD-11 (World Health Organization, 2022), we were therefore unable to measure PGD criteria as defined in ICD-11.

2 The standardized significant (*p* < 0.05) cross-lagged effects for PGD levels in parents predicting subsequent PGD levels in adult children varied between 0.05 and 0.06.

## Supplementary material

The supplementary material for this article can be found at https://doi.org/10.1017/S0033291723001101
