# Supplementary material for: Grief is a family affair: examining longitudinal associations between prolonged grief in parents and their adult children using four-wave cross-lagged panel models
Source: Psychol Med. 2023 May 8;53(15):7428–34. doi: 10.1017/S0033291723001101 (PMC10719676; doi:10.1017/S0033291723001101)
Supplement: Lenferink and O'Connor supplementary material [file S0033291723001101sup001.docx]

**Supplemental Materials**

**Table S1.** *Fit indices for cross-lagged model in parent-child dyads (N = 257)*

|  | **ꭕ^2^** | **df** | **CFI** | **TLI** | **RMSEA** | **SRMR** | **SA-BIC** | **AIC** |
| --- | --- | --- | --- | --- | --- | --- | --- | --- |
| Model 1 | 72.18 | 12 | 0.959 | 0.908 | 0.140 | 0.041 | 10072.13 | 10060.01 |
| Model 2 | 77.80 | 16 | 0.958 | 0.929 | 0.123 | 0.055 | 10068.23 | 10157.00 |
| **Model 3** | **82.80** | **20** | **0.957** | **0.943** | **0.111** | **0.060** | **10063.71** | **10054.62** |
| Model 4 | 111.66 | 23 | 0.940 | 0.930 | 0.122 | 0.128 | 10085.45 | 10077.49 |

*Note.* Model 1 is without constrains, Model 2 is with constraining autoregressive paths, Model 3 is with constraining autoregressive and cross-lagged paths. Model 4 is with constraining autoregressive, cross-lagged paths and paths between PGD at the same wave. The most parsimonious model is shown in bold. AIC = Akaike information criterion; CFI = comparative fit index; df = degrees of freedom; RMSEA = root mean square error of approximation; SA-BIC = sample-size adjusted Bayesian information criterion; SRMR = standardize root mean square residual; TLI = Tucker-Lewis index.

**Table S2.** Unstandardized estimates and concurrent associations for cross-lagged model when constraining autoregressive and cross-lagged paths in parent-child dyads (N = 257)

| **Autoregressive paths** | **B (SE)** |
| --- | --- |
| PGD in adult children | 0.748*** |
| PGD in parents | 0.769*** |
| Constrained cross-lagged paths |  |
| PGD in adult children 🡪 PGD parents | 0.045 |
| PGD parents 🡪 PGD in adult children | 0.051* |
| Concurrent associations |  |
| PGD in adult children W1 and PGD in adults W1 | 0.335*** |
| PGD in adult children W2 and PGD in adults W2 | 0.074 |
| PGD in adult children W3 and PGD in adults W3 | -0.025 |
| PGD in adult children W4 and PGD in adults W4 | 0.141 |

Note. PGD = B = regression coefficient; prolonged grief disorder; SE = standard error; W = wave. *** p < .001, ** p <.01, * p <.05.
